# On the Influence of Spatial and Value Attentional Cues Across Individuals

**DOI:** 10.5334/joc.229

**Published:** 2022-06-24

**Authors:** Kelly G. Garner, Michelle Lovell-Kane, Luke Carroll, Paul. E. Dux

**Affiliations:** 1 The University of Queensland, Saint Lucia, AU

**Keywords:** incentive value, endogenous, attention, counterfactual, reward

## Abstract

The visual world provides a myriad of cues that can be used to direct information processing. How does the brain integrate predictive information from disparate sources to modify visual priorities, and are combination strategies consistent across individuals? Previous evidence shows that cues predictive of the value of a visually guided task (incentive value) and cues that signal where targets may occur (spatial certainty) act independently to bias attention. Anticipatory accounts propose that both cues are encoded into an attentional priority map, whereas the counterfactual account argues that incentive value cues instead induce a reactive encoding of losses based on the direction of attention. We adjudicate between these alternatives and further determine whether there are individual differences in how attentional cues are encoded. 149 participants viewed two coloured placeholders that specified the potential value of correctly identifying an imminent target. Prior to the target’s presentation, an endogenous spatial cue indicated the target’s likely location. The anticipatory and counterfactual accounts were used to motivate parametric regressors that were compared in their explanatory power of the data, at the group level and on data stratified by a clustering algorithm. Clustering revealed 2 subtypes; whereas all individuals use spatial certainty cues a subset does not use incentive value cues. When incentive value cues are used their influence reflects a counterfactual loss function. The data support the counterfactual account and show that theories of motivated attention must account for the non-uniform influence of incentive value on visual priorities.

## 1 Introduction

Humans are good at exploiting predictive relationships between events to prioritise goal-relevant sensory data. For example, we learn quickly that the sound of a certain vehicle means that we can look for a loved one to enter our home, or that a red envelope poking out from the mailbox signals a potentially cheerful card inside. Yet how such predictive sensory cues are used to guide behaviour vary widely given the motivational state of the individual. Although a central and well studied tenet of many cognitive, computational and neurobiological theories of visual information processing is that competition for high-level representation is biased by predictive information pertaining to goal-relevant outcomes (*visual prioritisation*, see Moore and Zirnsak 2017; L. Itti and Koch 2001; Buschman and Kastner 2015, for reviews), far less is known about whether disparate sources of predictive information bias visual priorities in the same way. How are such cues combined, if at all? Do individuals differ in how they leverage predictive relationships between sensory cues and their consequent outcomes to form biases? Here we seek to understand how the brain encodes cues that carry information about potential reward outcomes (incentive value), and the extent to which this differs from how the brain uses cues that signal the probable location of task-relevant target information (spatial certainty). We also seek to understand whether individuals combine such cues in a homogeneous manner, or whether heterogeneity exists in the formation of visual priorities.

A large number of studies have shown that visual priorities can be modified by the incentive value signalled by sensory cues (see Le Pelley et al. 2016, for a review). Such studies typically teach participants to associate a simple cue such as a colour circle with a potential reward value such as a cash payout. In visual search tasks performance is better or worse when high value cues are presented as targets or distractors respectively (e.g. Anderson, Laurent and Yantis, (2011); Chelazzi et al (2014); Raymond and O’Brien (2009)). Similarly, it has been long established that performance is influenced in an analogous manner when sensory signals predict the spatial location where a target is likely to appear (Posner 1980). For example, target identification is faster when a preceding arrow points to the upcoming target location relative to when the arrow points away (see Chica et al. 2014; Carrasco 2011, for reviews). Although these two sources of expectation have comparable influences on visual priorities, i.e. improved performance at a signalled location at the expense of performance at other locations, a growing number of studies suggest that the influence of spatial and incentive value expectations are exerted via independent mechanisms (Stankevich and Geng 2014; Garner, Bowman, and Raymond 2021; Kim and Anderson 2021; Le Pelley et al, 2022). For example, Garner et al (2021) parametrically manipulated spatial certainty by adjusting the reliability of a central spatial cue, and pit these cues against high and low value target and distractor locations. They found an additive influence of each expectation (spatial certainty and incentive value) even when reward outcomes were structured to make additivity a suboptimal strategy, suggesting an independent influence. What remains to be determined is why two expectations that have such comparable effects upon visual prioritisation may be manifest independently.

One possibility, stemming from computational models of physical salience (Itti and Koch, 2000), is that differing expectations are encoded via independent filters, and a weighted summation is applied across filter outcomes to form an attentional priority map, where peaks reflect the points of visual priority (see also Failing and Theeuwes 2018; Awh, Belopolsky, and Theeuwes 2012). The consequence is that a scene is the sum of its visual parts (Beyeler et al. 2019). To wit, spatial certainty and incentive value are each encoded by unique filters prior to target onset, and their summed values are leveraged to build an *anticipatory* prioritisation of locations in the visual scene. We label such accounts as anticipatory, as they reflect an encoding of the cue stimuli that occurs prior to the onset of the target stimulus, thus these mechanisms act to bias information processing towards locations where the upcoming target may or may not appear. Specifically, the proposed mechanisms are anticipatory towards the upcoming target.

An alternate possibility is that spatial certainty and incentive value confer an independent influence on visual prioritisation because they are leveraged at distinct stages of information processing (Sternberg 1969). It is well established that symbolic spatial cues modulate visual prioritisation prior to target onset (see Desimone and Duncan 1995; Carrasco 2011; Buschman and Kastner 2015, for reviews). Previous studies finding an independent influence of incentive value and spatial certainty (e.g. Garner, Bowman, and Raymond 2021; Stankevich and Geng 2014) have pitted high and low incentive value cues between potential target and distractor locations. Importantly, although each value cue provided information about the reward incentive available should the target appear there, the actual reward available on a given trial is unknown until the target appears. Therefore the information offered by competing incentive value cues changes at target and distractor onset, where the distractor location becomes nullified with respect to potential reward incentive. Specifically, as long as the location of the target is detected, the participant already knows the only reward value they can possibly receive on that trial, irrespective of the actual feedback given at the end of the trial. Thus it is possible that the influence of incentive value on performance at least in part reflects a value update signal deployed after target onset. We do not refer to this an *anticipatory* prioritisation as it is deployed after target onset and is not used to bias information processing towards the upcoming target.

What information are cue encoding mechanisms sensitive to before target onset? The influence of spatial cues on the anticipatory prioritisation of potential target locations has consistently been shown to be mediated by the experienced reliability of the cue (Prinzmetal et al. 2015; Vossel et al. 2015; Lanthier et al. 2015; Garner, Bowman, and Raymond 2021). This implies that the brain implements an encoding scheme based on *selection history*, where the extent to which the cue information is leveraged to influence visual priorities is proportional to the expected value of the cue (e.g. Mackintosh 1975; Le Pelley et al, 2016; Failing and Theeuwes 2018). Comparably, it has been proposed that a *relative value* function underlies the influence of incentive value on visual priorities, where the expected value (EV) is computed for each cue, and each EV is normalised by the set of currently available expected values (Stănişor et al. 2013). If it were found that incentive value cues were encoded using such a relative value function, this would tie their encoding closely to that of spatial certainty, lending weight to the notion that both spatial and incentive value cues are encoded in a similar fashion into an anticipatory priority map (Stankevich and Geng 2014; Stănişor et al. 2013). Conversely, it has been proposed that incentive value cues may be encoded via an alternate anticipatory mechanism that leverages the *motivational* value offered by summing across all reward values that are signalled as available on a given trial (see Stănişor et al. 2013; Sawaki, Luck, and Raymond 2015; Manohar et al. 2017). In this case, performance is better when more high incentive values are on offer. Finding that incentive value cues are encoded using a *motivational* function would still place their influence as anticipatory, but would suggest that in contrast to spatial cues, their influence is generalised across the visual field.

How might incentive value cues be encoded if they instead reflect an update in response to the resolution of unknown reward outcomes that occurs at or after target onset, rather than an *anticipatory* bias towards upcoming target locations? In order to understand how the visual system may respond to incentive value information once the target has onset and likely outcomes are known, we leverage insights from statistical theory regarding causal inference (e.g. Pearl 2009). Specifically, these models suggest that one solution for determining causal contingencies is the representation of outcomes for both current choices and for the alternatives that were not currently taken (i.e. *counterfactual* representations). There is emerging evidence that such models may serve useful for understanding how the brain performs reasoning and decision-making. For example, fMRI activity in medial prefrontal cortex has been shown to correspond to the availability of counterfactual feedback in a value-based decision making task (Pischedda, Palminteri, and Coricelli 2020), and fMRI activity in prefrontal cortex corresponds to what would be expected if this region generates prediction errors that are proportional to the probability of reward gain for the best unchosen option (Boorman, Behrens, and Rushworth 2011). Collectively these findings imply the brain simulates the outcomes that can be gained from unchosen options in order to inform future decisions (Fouragnan et al. 2019). Here we test whether the influence of incentive values on visual priorities could reflect such a counterfactual update signal.

As mentioned above, the influence of expectations on visual priorities may also vary according to the current motivational state of the individual. Indeed, it is easy to imagine that all three of the above encoding mechanisms could be used to leverage the influence of incentive value, depending on the strategy implemented by the individual. This accords with recent theoretical assertions that the combinatorics of the visual world are so numerous that it is unlikely that a single solution exists for determining what sensory data should be prioritised with respect to ongoing expectations, and that individuals are likely to come up with one of a subset of solutions that best fits their own cost functions (Tsotsos et al. 2021). The over simplification of modelling cognitive mechanisms using one-size-fits-all assumptions has been demonstrated recently in the study of response inhibition. Rouder and Haaf (2021) showed that performance on tasks that are assumed to universally induce response conflict are best explained by models that allow conflict effects for some individuals and facilitative effects for others. This supports the notion that qualitatively different responses are present in the population. Here we address this issue in the context of when expectations influence visual priorities. Specifically, we seek to determine whether qualitative subtypes exist when spatial certainty and incentive value expectations are available to bias visual priorities.

We sought to determine whether spatial certainty and incentive value influence visual priorities by comparable or distinct mechanisms. Specifically, we expected spatial certainty to influence visual prioritisation in a manner that reflects sensitivity to selection history and we sought to determine whether incentive value cues confer their influence via a comparable mechanism (relative value), via a generalised anticipatory mechanism (motivation), or via a distinct counterfactual encoding mechanism. To this end we used each theoretical account to motivate a parametric regressor to predict performance on an adapted paradigm that pits spatial certainty against incentive value cues in a simple visual discrimination task (Garner, Bowman, and Raymond 2021). These parametric predictions were used in two ways. First, they were used to motivate a qualitative description of expected performance differences between conditions that were tested using a standard NHST approach. Second, the parametric regressors were used to predict performance using linear-mixed effects (LME) models and model comparisons were performed to determine which account best explained the observed data. Next we sought to determine whether qualitative subtypes exist in the use of spatial certainty and expected value cues. We therefore applied clustering algorithms to the data to determine the presence or absence of subtypes. The same LME models were then compared within each identified cluster to test whether each group used comparable or divergent strategies when employing spatial certainty and incentive value cues. To preempt the results, we confirm that the influence of spatial certainty is mediated by a selection history mechanism whereas incentive value cue use was best explained by our counterfactual encoding model. Two subtypes of individuals were identified in the data; whereas all individuals used spatial certainty cues, a subset of participants did not use incentive value cues to bias visual priorities. Those that did use incentive value cues were best explained with the counterfactual model. Collectively our data show that spatial certainty and incentive value cues are leveraged by distinct mechanisms to influence visual priorities, and that models of motivated attention need to account for qualitatively differing responses to incentive value cues.

## 2 Method

All data are available at UQ eSpace.^1^ Custom software for task presentation and analysis are available on Github.^2^

### 2.1 Participants

As per our pre-registration criteria, we sought to collect 150 complete datasets in order to have sufficient data to detect clusters should they be present (Hair, Black, and Babin 2010), while being achievable within a given data collection period for pragmatic reasons. A total of 154 participants were recruited for the study. Of these, 2 datasets were lost due to experimenter error, 2 participants were unable to meet the accuracy criteria to pass the first phase of the task (see below), and 1 data set was lost owing to a hardware malfunction. Of the remaining 149 participants retained in the study (115 right handed, mean age = 23.5 y, sd = 7.3), 107 reported being female sex, 42 reported being male sex. All procedures were cleared by the Human Research Ethics Committee at the University of Queensland, and were conducted in accordance with the National Statement on Ethical Conduct in Human Research.

### 2.2 Procedure

**Figure 1:**
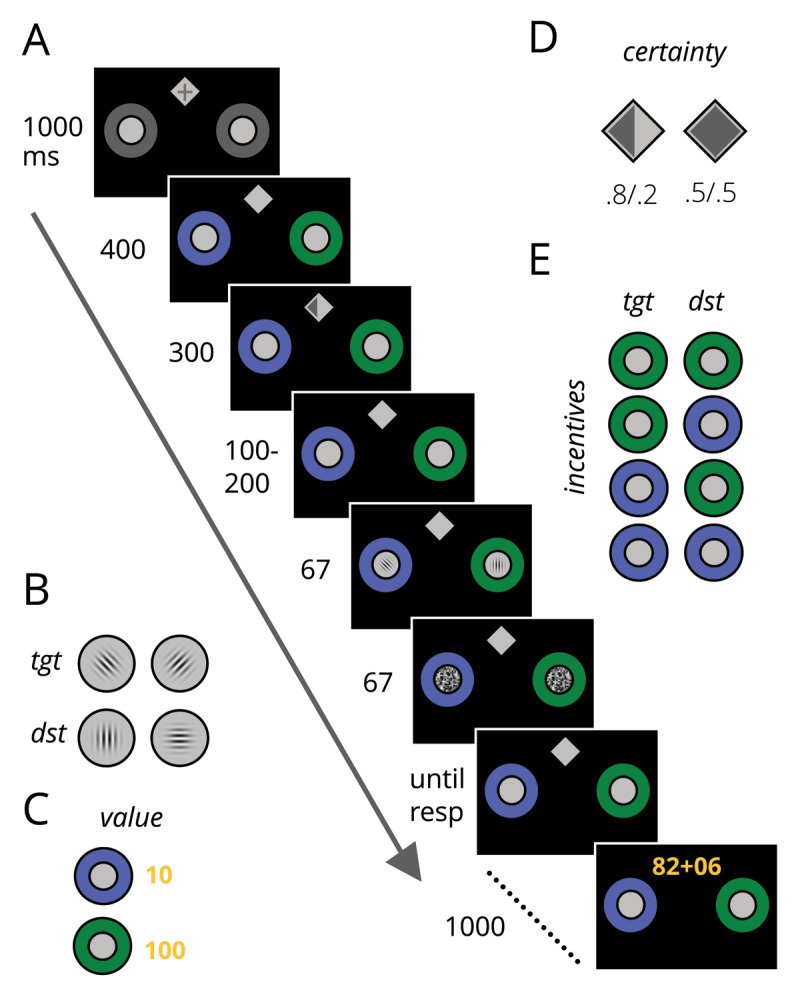
Experimental task. A) An example trial. Participants reported whether the target gabor was oriented clockwise or counterclockwise (B) the distractor was a gabor presented on the cardinal axis. C) Incentive value cues offered high (100) or low (10) point values. D) Spatial certainty cues were informative (p = .8) or non-informative (p = .5) regarding the upcoming target location. E) Incentive value cues were presented using 4 different configurations. tgt = target location, dst = distractor location, ms = milliseconds.

All tasks were run with custom code written using Matlab 2016b and Psychtoolbox v3.0.14 (Brainard 1997; Pelli 1997) on a Mac Mini computer (Late 2014 2.8 GHz Intel Core i5, OSX High Sierra v 10.13.6), and displayed using a ASUS VG248 monitor. Participants were seated from the monitor at an approximate viewing distance of 57 cm, and completed four stages; target learning, spatial cue learning, incentive value cue learning, and the experimental task. Figure 1 shows a depiction of the experimental task.

### 2.3 Target learning

Participants viewed a white fixation cross (RGB: 255, 255, 255) that subtended a diameter of 1° visual angle, with a line-width of 0.8°. The fixation cross was centrally presented on a black background (RGB: 0, 0, 0). Two dark grey rings (RGB: 114, 114, 114), with an inner diameter of 2°, served as placeholders for upcoming target and distractor locations, and were simultaneously displayed –/+ 3.2° from the vertical meridian, and 4.8° below the horizontal meridian. After a randomised interval lasting between 1700–1800 ms, two gabors were presented (gaussian weighted sinusoidal gratings), one over each dark grey circle. Each gabor was 3° in diameter. The target gabor was rotated –/+ 45° and the distractor gabor was presented on one of the cardinal axes. Both gabors were presented for 67 ms. Participants were instructed to indicate whether the rotated gabor was presented clockwise or counterclockwise relative to the vertical meridian using the ‘f’ and ‘j’ keys, using the two index fingers on the home keys of a standard QWERTY keyboard (response mapping was counterbalanced across participants). Location of the gabors (left or right), orientation of the distractor gabor (vertical or horizontal) and tilt of the target gabor was fully counterbalanced across trials. Contingent on the participant’s response, the message ‘Correct :)’ or ‘Incorrect :(’ was displayed in white text on the centre of the screen for 1000 ms. After the feedback message, the placeholders were removed and a dark grey cross, subtending 2° with a line width of 1° was centrally presented for 1000 ms. Trials continued until the participant scored >90% correct across the previous 20 trials. Next, the contrast of the gabors were titrated to yield ~75 % accuracy for each participant using the QUEST staircasing procedure (Watson and Pelli, 1983). The trials unfolded as described above with the following changes; subsequent to the target display, masks were presented over each gabor for 67 ms. Masks were made of noise patches which were a gaussian weighted square of random pixel values; generated from between the background colour to white. A broad range of contrast values were tested in the first 32 trials, which were titrated for each location over the subsequent 32 trials. The resulting contrast values were used for the remainder of the study. During this stage, participants did not receive feedback on their performance, as per explicit instruction. Instead of the feedback message, a dark grey period was presented centrally for 1000 ms.

### 2.4 Spatial cue learning

The spatial cue learning phase was the same as above with the following differences; each trial began with a centrally presented light grey diamond (RGB: 191, 191, 191), subtending 1.6°. A dark grey fixation cross (as above) was presented inside the diamond for 1000 ms. After this, the fixation cross was removed and the two dark grey rings appeared as before. After 400 ms, a dark grey spatial cue was presented within the diamond for 300 ms. The spatial cue was either directional or bidirectional. Directional cues appeared as a dark grey half fill of either the left or right side of the diamond. Directional cues validly signalled the location of the upcoming target gabor on p = .8 of all trials. The bidirectional spatial cue appeared as a full fill of the diamond, indicating that the target was just as likely to appear in the left placeholder as the right placeholder. The target and distractor gabors were presented after a random delay between 100–200 ms after cue offset. During this phase, participants received performance feedback as described above. Participants were explicitly instructed about the cue-target contingencies. Participants completed 3 blocks of trials, 1 with the left directional cue, 1 with the bidirectional cue, and 1 with the right directional cue. The bidirectional cue block contained 32 trials (16 with the target on the left). Directional cue blocks contained 40 trials (32 validly cued, 8 invalidly cued).

### 2.5 Value cue learning

The value cue learning phase occurred as above with the following differences: For each trial, one of the dark grey rings was presented in one of two colours (RGBs: 82, 95, 186; or 0, 130, 0). Participants were informed that each colour predicted how much reward was available on that trial, given they got the task correct. One colour signalled a high reward value, whereas the second signalled a low reward value. Regardless of reward value, if a target appeared in that ring then reward was available for p = .8 of trials, with no reward being attained on the remaining trials (p = .2). The participant was informed explicitly about the colour reward mappings. Colour to value assignment was counterbalanced across participants.

Across all trials (in this stage of learning), only a fixation cross was presented inside the grey diamond. Furthermore, the target always appeared in the one coloured ring (whereas the distractor appeared within the grey ring). Participants were explicitly informed that in the next stage of the task, both rings would be coloured and the directional cues would be reintroduced, therefore it still remained an optimal strategy to keep eyes trained on the central fixation cross.

For correct responses, the exact reward value on any given trial was calculated by measuring the response time of the participant as a proportion against a predefined interval between 350–850 ms. This was used to compute a proportion of the reward value (50 on high value trials, 5 on low value trials), that was added to the base level of reward available on that trial (50 on high value trials, 5 on low value trials). Therefore, on high value trials, participants could receive between 50 and 100 points, whereas on low value trials they could receive between 5 and 10 points, depending on their response speed. The points scored on that trial were presented subsequent to the participant’s response as yellow (RGB: 255, 215, 0) alphanumerics in the centre of the screen, next to the reward total accrued up until that trial and a plus sign. If the participant responded incorrectly, their previous score was presented centrally on the screen in grey alphanumeric characters for 1000 ms.

Participants completed 16 trials with each value cue before moving on to the full experimental task.

## 3 Experimental task

The full experimental task combined the above elements together in the following way: at trial onset, a grey fixation cross was presented inside the white diamond, along with the two coloured rings. After 400 ms, one of the spatial cues was presented for 300 ms. After a random interval between 100 and 200 ms after spatial cue offset, the target and distractor were presented for 67 ms, followed by the masks for 67 ms. Feedback was presented subsequent to the participant’s response as outlined above, followed by the grey fixation which was presented for 1000 ms. Target location (left vs right) was fully counterbalanced across trials. Overall participants completed 2 blocks each consisting of 360 trials; of these 200 contained a directional cue (160 valid/40 invalid), and 160 contained a bidirectional cue. Each of the cue conditions (invalid/bidirectional/valid: p (target location|cue) = .2/.5/.8) were equally divided into four different value conditions; low value target location/low value distractor location [lT/lD], lT/hD, hT/hD, hT/lD. Each block of counterbalanced trials were presented in pseudorandom order, allocated using the Matlab *randperm* function. The total trial number (720) was divided into 36 blocks of 20 trials. Participants were instructed to respond to the target gabor as accurately and quickly as possible in order to win points.

### 3.1 Data analysis and statistical design

As per our pre-registration criteria, participants scoring < 60 % across all conditions were excluded from the analysis. This resulted in the exclusion of two participants (remaining N = 147). RT outliers for correct trials were defined as < 250 ms, and those > 2.5 standard deviations above the median for each subject x spatial certainty x relative value condition. QQ plots of the remaining data revealed that the RT distributions deviated from normality, so we performed subsequent analyses on each individual’s median RT scores.

#### 3.1.1 Theoretical models

**Figure 2:**
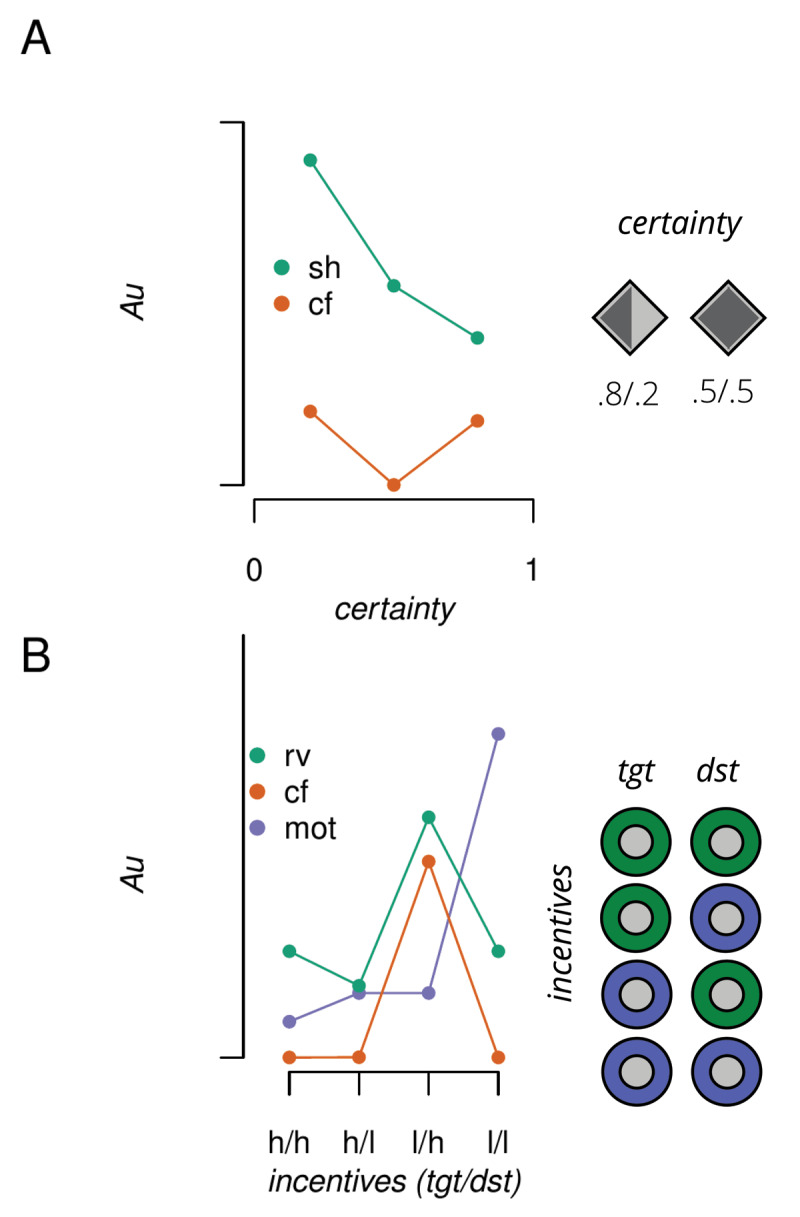
Theoretical predictions for the influence of spatial certainty and incentive value cues. A) Predicted RTs in arbitrary units (Au) given a selection-history (sh) or counterfactual (cf) encoding of the spatial cue. B) Same as A but for the influence of incentive value cues given relative value (rv), cf or motivational (mot) encoding. tgt = target location, dst = distractor location, h = high value, l = low value.

Given preceding information about where a target is likely to appear (spatial certainty), or how much value a potential target is worth, given its signalled spatial location (incentive value), there are multiple potential encoding strategies that could be used to facilitate visual information processing. We seek to use theoretical accounts of how the brain encodes spatial certainty and incentive value cues to generate parametric regressors to predict performance. We then compare which regressors provide a better account of the observed data. The parametric regressors are depicted in Figure 2.

To make predictions about performance given differing theoretical encoding strategies, we first take advantage of the finding that RT follows the Hick-Hyman law of decision-time (Hick 1952; Hyman 1953), and that this law has been shown to also predict behaviour in spatial cueing paradigms (Garner, Bowman, and Raymond 2021; Prinzmetal et al. 2015). This law states that RT shares a monotonic relationship to the information offered by a sensory cue:


1 R{T_x} = \beta \;{\rm{log(}}\frac{1}{{{p_x}}} + 1{\rm{)}}

where RT is response time for a given target *x, p* is the probability of the event, and 1 reflects the option to not respond. Note that for the counterfactual encoding theory outlined below, it is sometimes possible that *p* = 0, so we modified the equation above to be a piecewise function, where in the case that p = 0, the first term is dropped from the model and the formula becomes *RT_x_* = *β* log(1) = 0.

Importantly, how *p* is calculated varies according to the set of events that a given encoding mechanism is tracking. An *anticipatory* account (Failing and Theeuwes 2018) poses that in the case of spatial certainty, *p* is equal to the experienced probability of the spatial cue (Le Pelley et al, 2016; Mackintosh 1975). Comparably, *anticipatory* accounts for the influence of incentive value suggest that *p* is computed by taking the expected value for each value cue, normalised by the set of expected values for that trial (Stănişor et al. 2013, *relative value*):


2 p = \frac{{{v_x}}}{{\mathop \sum \nolimits_{x = 1}^X {v_x}}}

where *v* is the value available at location *x*, for the set of *X* locations. As can be seen in Figure 2 panel B, this account predicts that performance is related to the proportion of the total available reward signalled by each location. Therefore RTs should be fastest when the target appears at a high value location paired with a low value distractor location (hT/lD), and slowest for the opposite scenario. Importantly, performance when both locations signal high incentive value (hT/hD) should be comparable to lT/lD, as each location (e.g. hT *or* hD) represents 50 % of the total reward signalled as available on that trial (i.e. is one of two value cues currently displayed). Moreover, performance on these two conditions should lie between that observed for the hT/lD and lT/hD conditions, analogous to what would be expected when a spatial cue offers no certainty about the upcoming target location (p (target|cue = .5)).

As noted above, in addition to the relative value model, it has been proposed that value may induce a widespread, motivational boost to visual information processing, proportional to the sum of signalled values for that trial (Stănişor et al. 2013; Sawaki, Luck, and Raymond 2015)) This *motivational* encoding model predicts that *p* from equation 1 is computed as:


3 p = \mathop {\sum \;}\limits_1^X {p_x}*{v_x}

This account predicts that performance will be fastest for hT/hD, slowest for lT/lD, with performance for the hT/lD and lT/hD conditions falling somewhere in between.

An *anticipatory* mechanism assumes that the agent can act on learned expected values about the world to bias sensory information processing. In order to build a model of causal relationships about the world, it is essential to track events that did not happen, e.g. what is the reward I would have attained if the target had appeared at location *a* instead of *b*, and how far is that from the best reward value currently on offer? To generate a model of such *counterfactual* encoding in the current task context, we adapted a counterfactual loss function suggested by Wachter et al (2017). In this scheme, possible losses (L) from both experienced and non-experienced events are tracked using the following loss function:


4 L\left({x,x^{\prime},y^{\prime}} \right) = {\left({x^{\prime} - y^{\prime}} \right)^2} + d\left({x,x^{\prime}} \right)

where *x’* is the predicted value of the counterfactual outcome, and *y’* is the desired outcome, which is set in advance as the maximum reward value available on a given trial. *d* is the distance between what happened (*x*) and *x’*. Thus, this function measures how far the predicted outcome of the counterfactual is from the desired outcome, and how far the counterfactual outcome is from the experienced event. The distance function *d* is the distance of the experienced event *x* from the counterfactual outcome *x’*, weighted with the inverse median absolute deviation (MAD) of each feature:


5 d\left({x,x^{\prime}} \right) = \left\{ \begin{array}{l}
{\rm{0}}\,\,\,\,\,\,\,\,\,\,\,\,\,\,\,\,\,\,\,\,\,\,\,\,\,\,\,\,\,\,\,\,{\rm{if\;}}\left({x - x^{\prime}} \right) = 0\\
\mathop \sum \nolimits_1^p \frac{{\left| {{x_j} - {{x^{\prime}}_j}} \right|}}{{MA{D_j}}}\,\,\,\,\,\,\,{\rm{if\;}}\left({x - x^{\prime}} \right) \ne 0
\end{array} \right.

i.e. it is the absolute median distance between all relevant feature values and the counterfactual outcome. Using this function, *p* from equation 1 is computed as:


6 p = L

This model predicts that performance is related to the loss accrued by not attaining the counterfactual outcome, given the maximum reward value available on that trial. Therefore performance should be comparable across hT/hD, lT/lD and hT/lD conditions, as on these trials the actual outcome was either equal to or better than the counterfactual outcome. In contrast, RTs should be selectively slowed for lT/hD trials, where the loss from the actual outcome (lT) was further from the best possible outcome (h) relative to the counterfactual outcome (hD), see Figure 2B.

#### 3.1.2 Statistical Design

**Qualitative Predictions** Two approaches were used to determine which theoretical models best accounted for the data. First, the pattern of predicted results allowed for qualitative statements to be made about anticipated differences between spatial certainty and incentive value conditions. Specifically, for spatial certainty, an anticipatory (selection history) model predicts that performance is directly proportional to the reliability of the cue, therefore RTs should be fastest for p (target|cue) = .8 conditions, next fastest for p = .5, and slowest for p = .2. (see Figure 2). (Note: we included a counterfactual regressor for the influence of spatial certainty as a null model against which to compare the anticipatory model). For incentive value, an anticipatory relative value model predicts that performance will be fastest in the hT/lD condition, next fastest and comparable for the hT/hD and lT/lD conditions, and slowest for the lT/hD condition. A motivational model, while still anticipatory, predicts that performance should be fastest for the hT/hD condition, next fastest for the lT/hD and hT/lD conditions, and slowest for the lT/lD condition. The counterfactual model instead predicts comparable performance for hT/hD, lT/lD and hT/lD conditions, and a selective slowing for lT/hD trials. Note that selective slowing in the presence of high value distractors has recently been observed in alternate paradigms using colour singletons as distractors (Watson et al, 2020) or valued (positive or negative) vs neutral colours in a dot probe task (Müller, Rothermund, and Wentura 2016). We shall return to how the current work complements and extends these findings in the discussion.

To test these qualitative predictions, RT and accuracy data were each subject to a 3 (spatial certainty) x 4 (incentive value configuration) repeated measures ANOVA. Statistically significant results were followed up using t-tests, to identify where found differences matched the qualitative predictions. The FDR correction was applied for multiple comparisons (q < .05). Bayesian t-tests were applied in cases where predicted differences were null and post-hoc t-tests did not find evidence to reject the null hypothesis using the BayesFactor package for R (Morey, Rouder, and Jamil (2015)).

**Quantitative Predictions** Second, we applied a quantitative approach by determining which of the theoretical regressors provided the best account for the data using a linear-mixed effects (LME) approach. To assess whether the influence of spatial certainty is best explained by an anticipatory or counterfactual encoding mechanism, the data (averaged over the incentive value factor) were entered into LME models using either the anticipatory or counterfactual predictions as regressors. LME models contained constant terms for each subject and for the subject x conditions interaction. Models were compared using AIC and BIC, and by examining model fits to the data. To determine whether the parametric regressors could reliably predict the data, the resulting parameter estimates were assessed for statistical significance using the Wald chi-square test as implemented in the Anova function from the car package (Fox and Weisberg 2018). The same procedure was applied to the incentive value data, with the additional fitting of the regressor generated using the motivational encoding account.

**Individual Differences** To test for the presence of qualitative subtypes, the data were subject to a clustering analysis. The key effects of interest were calculated for each participant. The size of the spatial cueing effect was computed by subtracting RTs for high certainty trials (p (target location| cue) = .8) from low certainty trials (p(target location| cue) = .2). The size of the influence of incentive value was computed by subtracting RTs from the hT/hD condition from the remaining conditions (hT/lD, lT/hD, lT/lD), yielding three scores. To determine the reliability of these measures within individuals, these scores were also computed using the data taken from odd and even trials and were subject to a correlation analysis, with higher correlation values being interpreted as reflecting greater reliability. The features (spatial cueing effect and 3 x incentive value effects) were scaled prior to being entered into the clustering analysis.

When using unsupervised algorithms it is important to ensure that the discovered data subsets do not reflect the result of an artifactual structure; i.e., the clustering solution should reflect a likely solution rather than something that has been forced onto the data. We applied two distinct clustering algorithms to protect against such a possibility and to validate our findings. First, we applied a K-means clustering algorithm. K-means clustering seeks to sort data points in such a way that each observation in a cluster is more alike to those within the cluster than observations in other clusters, by minimising the squared Euclidean distance to a given cluster centroid. We tested cluster solutions between 1 and 10, with each solution performed over 10000 different random initialisations (Hofmans et al. 2015), to avoid ending in a local minimum. The optimal number of clusters was based on the largest relative decrease in the sum of squared Euclidean distances, the mean silhouette width (a measure of how similar datapoints are to their own cluster relative to other clusters), and by maximisation of the Dunn index (DI) (Dunn 1974). Second, we applied an OPTICS algorithm which uses discontinuities in distances between data points to identify clusters (reachability distance). One of the advantages of the OPTICS algorithm is that it allows identification of both clusters and outliers; the latter being points that lay beyond the bounds of any given cluster and that do not share that space with the minimum number of data points required to constitute a cluster. Following convention we defined this minimum cluster membership as the number of features plus 1. Using these convergent clustering techniques we identify two subtypes in the data, however whether these are interpreted as two distinct clusters or one cluster and a group of outliers is dependent on the specific algorithm employed. Importantly, the subsequent behavioural differences observed between groups was consistent regardless of the specific algorithm used to assign cluster membership.

To validate the cluster subtypes, and to determine any differences in the extent to which the 2 groups used spatial certainty and incentive value cues, the behavioural data were subject to an ANOVA as defined above, with the additional group factor, making it a 2 (group) x 2 (spatial certainty) x 4 (incentive value) mixed ANOVA. Observed differences were followed up using the procedure defined above. Lastly, to see if the groups differed in how they encoded the cues, the data were subject to the same LME models, this time applied to each group separately.

## 4 Results

### 4.1 Testing the influence of spatial certainty and incentive value

**Figure 3:**
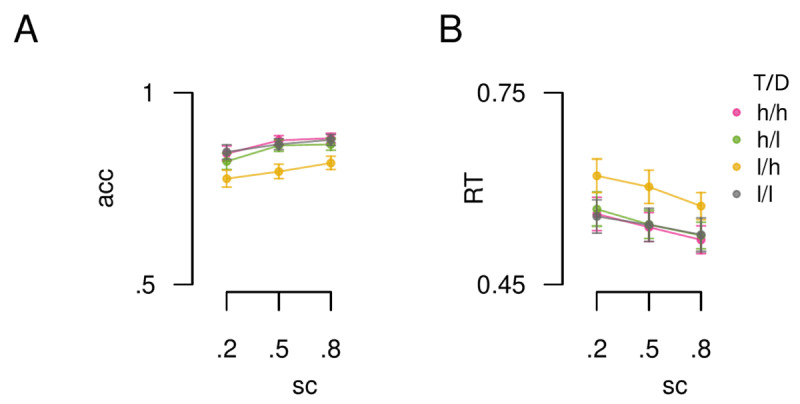
Influence of spatial certainty and incentive value. A) Group mean accuracy data plotted by spatial certainty (x-axis, sc) and incentive value condition (lines). B) RT data plotted in the same format as panel A. T = target location, D = distractor location, h = high value, l = low value. Error bars reflect standard error of the mean (SEM).

As can be seen in Figure 3, spatial certainty showed a statistically significant influence on RTs (F(1.21, 176.10) = 39.53, \eta _p^2 = 0.21, p = 1.43e–10), as did incentive value (F(2.17, 316.38) = 38.31, \eta _p^2 = 0.21, p = 1.37e–16). In line with previous observations that the influence of both factors are mediated by distinct mechanisms (Garner, Bowman, and Raymond 2021), the interaction was not statistically significant (F(3.89, 568.38) = 2.08, \eta _p^2 = 0.01, p = 0.08).

The influence of spatial certainty was in line with previous observations that performance scales with the likelihood of the target location, given the spatial cue (Garner, Bowman, and Raymond 2021; Prinzmetal et al. 2015; Vossel et al. 2015). Specifically, RTs were on average 21 ms faster (95% CI [16, 25]) for p (tgt location| cue) = .8 relative to p = .5. Moreover, RTs to the p = .5 condition were on average 19 ms faster (95% CI [10, 28]) than RTs to the p = .2 condition.

In line with the notion that a counterfactual encoding mechanism mediates the influence of incentive values on visual prioritisation, there was a selective slowing of RTs to the lT/hD condition relative to the 3 remaining conditions. Specifically, RTs were slowed by 59 ms (95% CI: [46, 71]) relative to the hT/hD condition (t(146) = 9.32, *d* = 0.77, p = 5.16e–16), by 56 ms (95% CI: [45, 67]) relative to the lT/lD condition (t(146) = 10.21, *d* = 0.85, p = 4.95e–18), and by 52 ms (95% CI: [39, 65]) relative to the hT/hD condition (t(146) = 7.91, *d* = 0.65, p = 1.17e–12). Comparisons between the remaining conditions did not reach statistical significance (all ps > 0.24). Importantly, post-hoc Bayesian t-tests showed evidence for the null that there was no difference between the lT/lD, hT/hD and hT/lD conditions (all *BF*_10_’s ≥ 0.23 +/– 0%).

As can be seen from the accuracy data in Figure 3, the results were not due to a speed/accuracy trade off. The accuracy data showed the inverse pattern to the RT data.

### 4.2 Group-level LME Modelling

**Figure 4:**
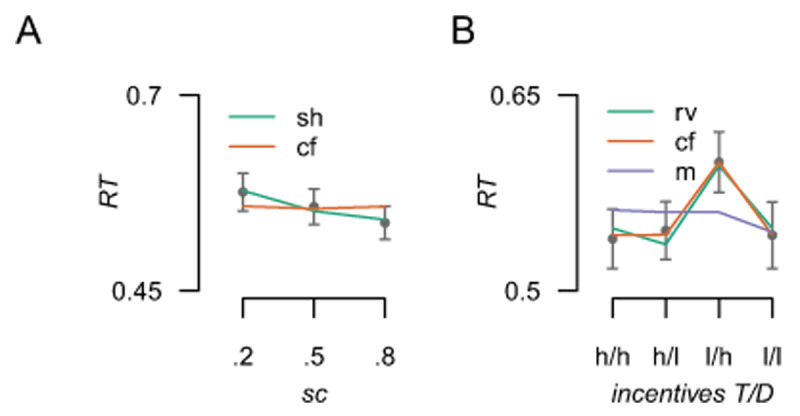
Model predictions plotted against the data for the influence of spatial certainty and incentive value. A) The influence of spatial certainty; the anticipatory (sh: selection history) and counterfactual (cf) model predictions plotted against the observed group average RT data (points). B) Predictions for the anticipatory (rv: relative value), counterfactual and motivational salience (m) models against the observed data. T = target location, D = distractor location, h = high value, l = low value. Error bars reflect standard error of the mean (SEM).

Next we determined which of our parametric regressors best accounted for the influence of each cue on performance. Here we predicted median RT’s, however the pattern of results was the same if we used an inverse efficiency measure (ie = {\textstyle{{RT} \over {acc}}}). Comparisons of LMEs showed that for spatial certainty, the *anticipatory* (selection history) model (*AIC*: –1221.13, *BIC*: –1196.59) provided a better account of the data than the *counterfactual* model (*AIC*: –1015.39, *BIC*: –990.86, see Figure 4. Moreover, Wald chi square tests showed that while the selection history regressor was a significant predictor of performance (*W_t_*(1) = 41.08, *p* = 1.46e–10), the counterfactual regressor was not (*W_t_*(1) = 0.52, *p* = 0.47).

In contrast, the influence of incentive value was best predicted by the *counterfactual* model (*AIC*: –1268.50, *BIC*: –1251.00, see Figure 4) over the *anticipatory* models (relative value: *AIC*: –1259.07, *BIC*: –1241.57; motivational encoding: *AIC*: –1174.10, *BIC*: –1156.60). Interestingly, all 3 regressors were able to predict performance at a level that was statistically significant (counterfactual model: *W_t_*(1) = 114.50, *p* = 1.01e–26, relative value: *W_t_*(1) = 102.75, *p* = 3.80e–24, motivational encoding: *W_t_*(1) = 7.46, *p* = 0.006), demonstrating the importance of model comparisons when using parametric regressors to predict performance. Indeed, this is perhaps unsurprising given that the predictions made by the relative value and counterfactual models share some similarities in the predictions they make. Importantly both the qualitative and quantitative comparisons yield support for the counterfactual account.

Having found that the influence of spatial certainty and incentive value cues are best accounted for by anticipatory and counterfactual encoding models respectively, we next determined whether individuals all conform to the same encoding solutions, or whether subtype solutions are present. Specifically, we clustered data into subtypes and determined which models best accounted for the performance of each subtype.

### 4.3 Reliability analysis

As we sought to understand whether systematic individual differences underlie the group average, it is important to ensure that the performance measures reflect a stable feature for each individual. To this end, we performed a reliability analysis on the features that were used in the clustering analysis. The size of the spatial certainty effect was defined as *SC* = *RT_p(target|cue = .2)_ – RT_p(target|cue = .8)_*. The size of the incentive value cueing effects were defined as follows: *IVi* = *RT_hThD_* – *RT_lTlD_, IVii* = *RT_hThD_* – *RT_lThD_, IViii* = *RT_hThD_* – *RT_hTlD_*. As can be seen from Table 1, all measures showed good reliability and were therefore deemed suitable for use in the clustering analysis.

**Table 1:** Reliability of the key behavioural effects.


EFFECT	R	DF	P

SC	0.696	145	1.29e–22

IVi	0.926	145	2.40e–63

IVii	0.908	145	1.18e–56

IViii	0.828	145	3.48e–38


### 4.4 Clustering analysis

**Figure 5:**
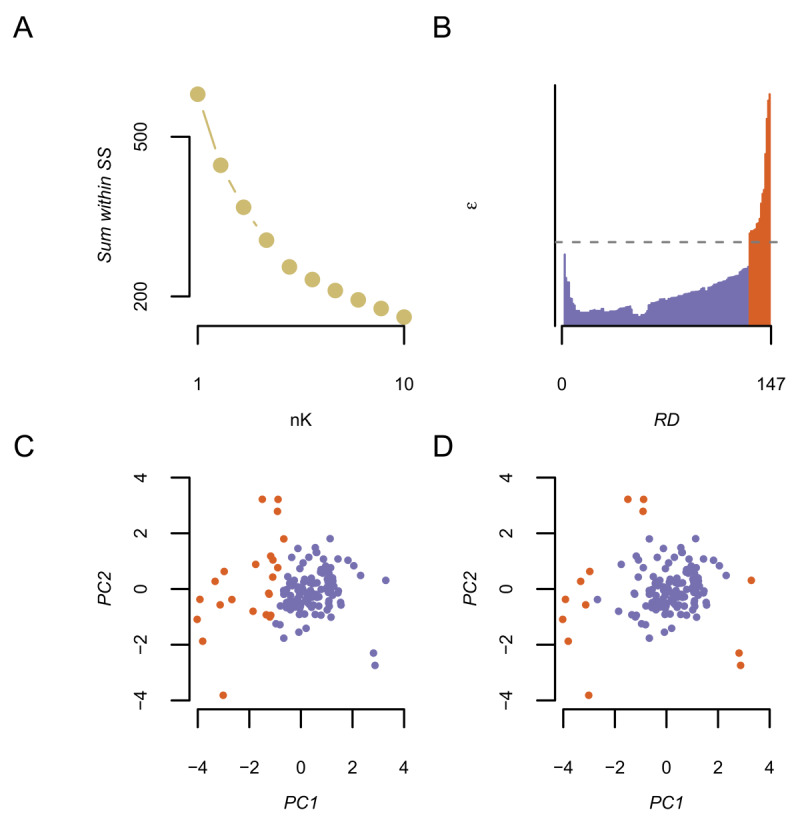
Sorting outcomes from application of k-means and OPTICS clustering algorithms. A) Sum of within sum of squares (SS) for each cluster solution (nK: x-axis) from the k-means algorithm. B) Data points ordered (x-axis) by Reachability epsilon distance (y-axis) by the OPTICS algorithm. C) The first two principal components of the feature space for the clustering analysis, where each participant is plotted as a point. Colour dentotes cluster group membership as found by the k-means algorithm. D) Same as C, except denoting group membership as found by the OPTICS algorithm. PC = principal component.

To determine whether subtypes could be observed in the data, the key effects (SC, IVi, IVii, IViii) were grouped using both a k-means and an OPTICS clustering algorithm. Prior to clustering the data were checked for multivariate outliers. One datapoint with a Mahalanobis distance of greater than 98 was less than 1% likely to have occurred by chance, given the degrees of freedom (146), and was removed from the analysis. However, the pattern of subsequent results was the same regardless. As can be seen in Figure 5 both algorithms identified two subtypes, but the nature of the subtypes differed. Specifically, the k-means algorithm favoured a 2-cluster solution (*N*_1_ =121, *N*_2_ =25) given the largest drop in within-SS occurred when moving from 1 to 2 clusters. Moreover comparison between this and the next best solution (3 clusters) showed that the DI was larger for the 2 cluster solution (*DI*_2_*_k_*: 1.06 > *DI*_3_*_k_*: 0.80), as was the average silhouette width (*SW*_2_*_k_* = 0.39 > *SW*_3_*_k_* = 0.23). This suggests two subtypes in the data. In contrast, the OPTICS algorithm favoured identification of 1 cluster (*N*_1_ = 131) and 15 outliers; the latter being too far to be classified as belonging to the main cluster, and also not close enough to a sufficient number of datapoints to be considered a second cluster. Given that the OPTICS solution appears potentially less likely to have produced misclassifications (see panels C & D of Figure 5), and the apparent lack of a clear inflection point in the kmeans elbow plot (see Figure 5) the remaining group comparisons are made using the grouping variable from the OPTICS algorithm. Importantly, the same pattern of group differences were found in the subsequent analysis, regardless of the specific clustering algorithm used to organise the data into subtypes. In summary, although both algorithms appear to favour classification of individuals into two categories, the OPTICS algorithm suggests that the data consists of one large group of individuals who performed comparably, and one smaller group of those who can not readily be classified into a particular type of performance.

### 4.5 Subtype comparisons

**Figure 6:**
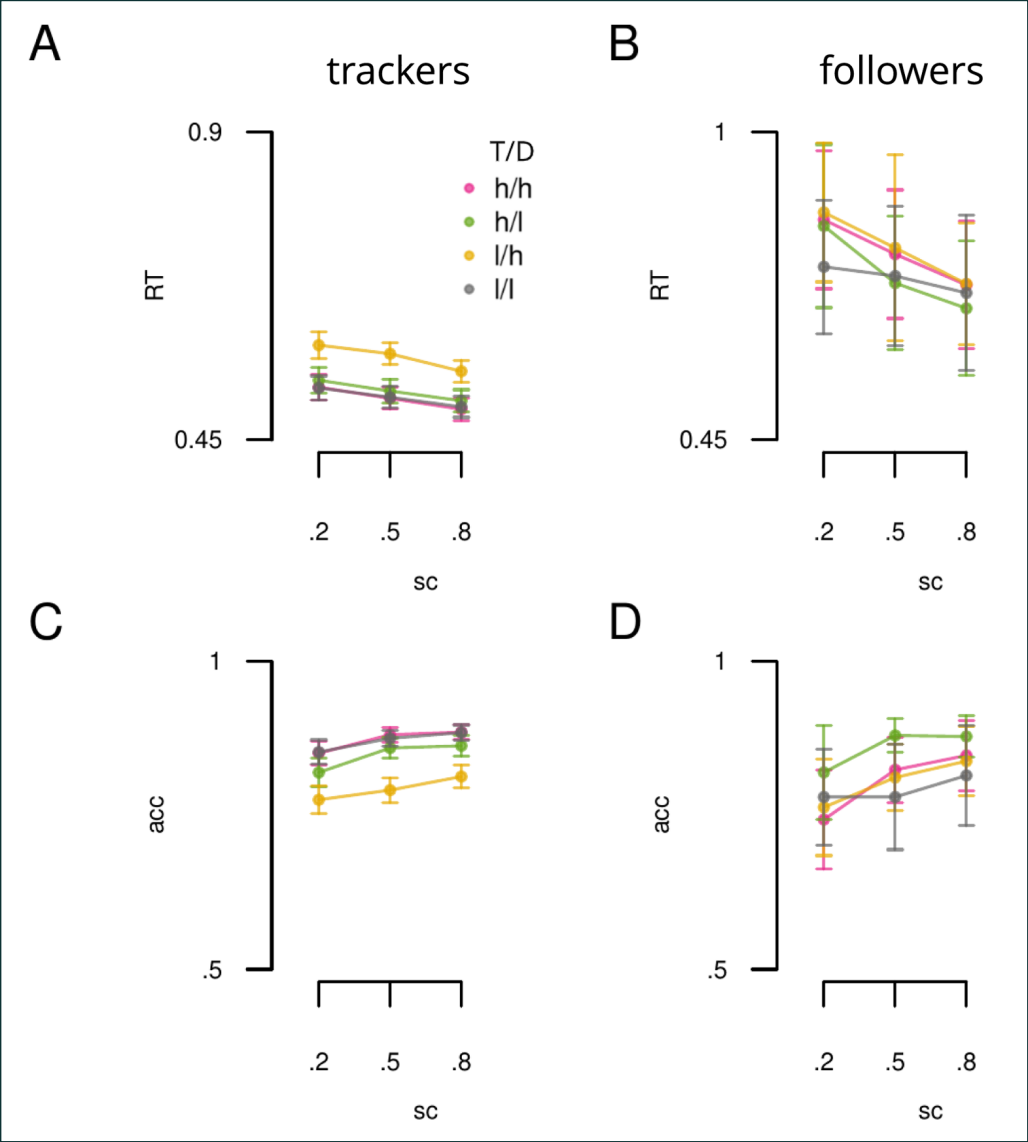
RT and accuracy data plotted separately for the 2 cluster groups. A) Showing RT data across spatial certainty (sc: x-axis) and incentive value (lines) for the trackers group (N = 131). B) Same as A, but for the followers group (N = 15). C) and D) Accuracy (acc) data plotted according to the same conventions as A and B. Error bars reflect SEM. T = target location, D = distractor location, h = high value, l = low value.

Having established two groups in the data (*N*_1_ = 131 and *N*_2_ = 15), we next sought to further validate this grouping by determining whether reliable differences could be observed between the two groups. Moreover we tested whether observed differences yielded insights into how individuals differ when using spatial certainty and incentive value cues. Given the disparity in group *N* we sought to make sure any group differences found were not due to an increase in type 1 error driven by inhomogeneity in group variances. For RT, the ratio of variances between groups was 5.84, thus use of an ANOVA test is likely to lead to inflated type 1 error (Blanca et al. 2018). Furthermore, previous simulation work shows that robust approaches (e.g. Welch test) may also lead to an increased rate of false positives when a lower *N*, such as we have in group 2 is combined with a ratio of variances such as is observed here (Wilcox, Char1in, and Thompson 1986). We were also unsatisified that standard transformations could sufficiently correct the data. For these reasons we sought to be conservative and only compared the groups on accuracy performance; as the ratio of variances was 1.52, suggesting that the ANOVA would be robust in this case (Blanca et al. 2018; Wilcox, Char1in, and Thompson 1986). Although we only analysed the accuracy data, both the RT and accuracy data are presented in Figure 6 for completeness. Note that this approach also carries the advantage that we are conducting follow up tests on a feature (accuracy) that we did not include in the clustering analysis, which strengthens the validation of the clustering analysis if group differences are found.

A 2 (group) x 3 (spatial certainty) x 4 (incentive value) mixed ANOVA showed a statistically significant group x incentive value interaction (F(2.10, 302.58 = 7.09, \eta _p^2 = 0.05, p = 7.97e–04). To break down this interaction, the average accuracy was computed for each incentive value condition and a one-way ANOVA was performed for each group separately. The first cluster group (*N*_131_) showed a statistically significant main effect of incentive value (F(2.08, 270.90 = 43.94, \eta _p^2 = 0.25, p = 8.81e–18). In contrast, this effect was not observed for the second group (*N*_15_; F(3.00, 42.00) = 1.30, \eta _p^2 = 0.09, p = 0.29).

A follow-up Bayesian ANOVA was applied to determine whether the absence of a statistically significant influence of incentive value was due to a genuine null effect, or whether the reduced *N* had resulted in insufficient power to detect an effect. The model containing a main effect of incentive value was compared to a null model containing only a constant term for each participant. The evidence was anecdotally in favour of the null hypothesis that there was no reliable influence of incentive value in this group (*BF*_10_ = 0.36, ± .12). While the large variance over the mean estimates (see Figure 6D) makes it difficult to definitively determine whether there may be a value effect that manifests differently in this group, the current data suggests that this may not be systematically the case. As the first group were using both spatial certainty and incentive value information, they shall now be referred to as ‘trackers’, analogous to an agent who monitors multiple environmental cues to find a target. The second group shall be referred to as ‘followers’, as they appear to have formed a strategy of just responding to a single source of information, namely they are opting to follow the spatial cue.

**Figure 7:**
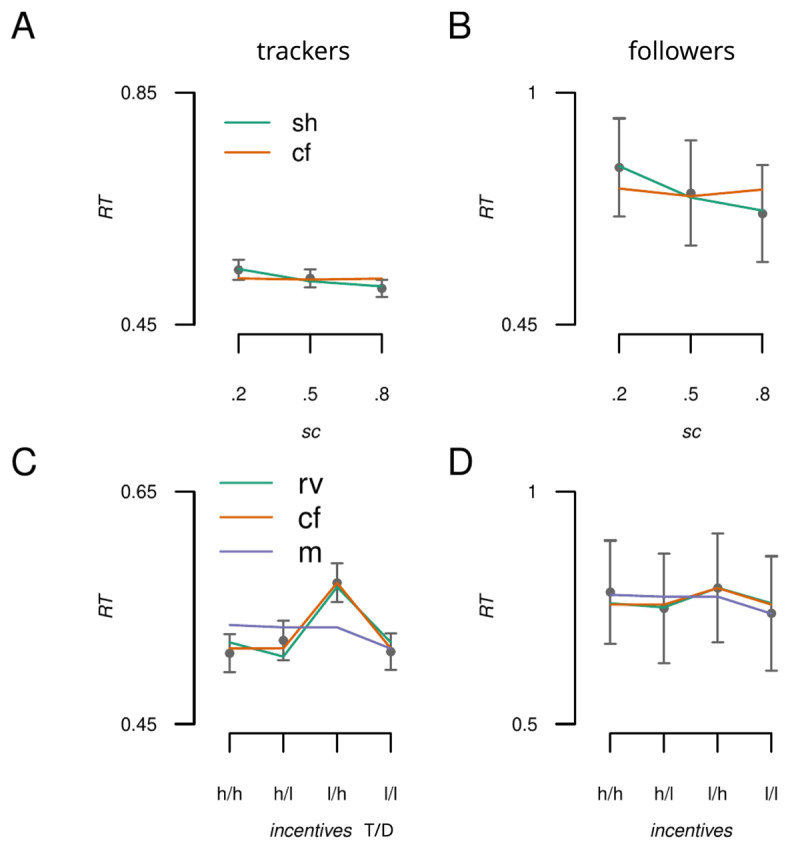
Model predictions plotted against the data for the trackers and followers groups. A) Response to spatial cues for the tracker group; anticipatory (sh: selection history) and counterfactual (cf) model predictions against the observed group average RT data (points). B) Same as A except for the followers group. C) Predictions for the anticipatory (rv: relative value), counterfactual and motivational salience (m) models against the observed influence of incentive values for the trackers group. D) Same as C but for the followers group. T = target location, D = distractor location, h = high value, l = low value. Error bars reflect standard error of the mean (SEM).

Next we asked whether both trackers and followers use the same encoding mechanisms when using spatial certainty and incentive value cues. We therefore applied the same LME models from above to the data from each group separately. The results are presented in Figure 7. For spatial certainty, the *anticipatory* model better explained the performance of both groups (trackers: *AIC*: –1400, *BIC*: –1376, followers: –53.2, *BIC*: –42.4), relative to the counterfactual model (trackers: *AIC*: –1240, *BIC*: –1216, followers: –26.3, *BIC*: –15.4). Moreover, Wald chi square tests showed that the selection history regressor was a significant predictor of performance for both groups (trackers: *W_t_*(1) = 73.7, *p* = 9.03e–18, followers: *W_t_*(1) = 5.83, *p* = 0.016).

For incentive value, trackers’ performance was best accounted for by the *counterfactual* model (*AIC*: –1641, *BIC*: –1616, see Figure 7) than by the *anticipatory* models (relative value: *AIC*: –1598, *BIC*: –1573; motivational encoding: *AIC*: –1413, *BIC*: –1388). Again, all 3 regressors were able to predict performance at a level that was statistically significant (counterfactual model: *W_t_*(1) = 159, *p* = 2.32e–36, relative value: *W_t_*(1) = 138, *p* = 7.60e–32, motivational encoding: *W_t_*(1) = 19, *p* = 1.32e–05). This was not the case for the followers group, where none of the regressors achieved statistical significance (all p’s > 0.11). We therefore do not draw inferences about model comparisons in this group.

## 5 Discussion

We sought to determine whether spatial certainty and incentive value influence visual priorities by comparable or distinct encoding mechanisms, and whether individual differences exist in how these expectations are configured to wield influence. 149 participants performed a simple discrimination for visual targets that were preceded by sensory cues that offered information about where the target was likely to appear (spatial certainty; arrows), and how much the task was worth should the target appear at a given location (incentive value; coloured placeholders). Testing of both qualitative and quantitative predictions revealed that while spatial certainty is used proportionally to the information offered by the cue (selection history), the influence of incentive value cues on performance is instead best predicted by a counterfactual encoding model. This model assumes that performance is related to counterfactual loss; specifically, performance is slowed when the target appears at a location that is lower in value than the best that is on offer (the counterfactual outcome). A negligible update (if any) is required when the target appears at a location that shares the same value as the best available, and so incentive value cues do not influence performance under these conditions. We also sought to determine whether individuals responded to spatial certainty and incentive value cues in the same way, or whether subtypes exist in the configuration of cue use. Clustering analyses showed convergent evidence for two types of respondents in the data; one group that used both spatial certainty and incentive value cues (trackers) and one that used only spatial certainty cues (followers). In corroboration with the group level analysis, trackers’ use of incentive value cues was best accounted for by a counterfactual encoding model. Therefore subtypes exist in terms of whether or not incentive value cues are adopted for visual priorities. However once adopted they appear to be consistently implemented using counterfactual encoding.

Previous observations of the independent influences of spatial certainty and incentive value cues have motivated the conclusion that both expectations are separately yet comparably encoded into an anticipatory priority map (Stankevich and Geng 2014; Garner, Bowman, and Raymond 2021). This is perhaps surprising given previous findings showing large overlap in the neurons that encode spatial and reward expectation information (Stănişor et al. 2013) and the presumed interactive relationships between top-down selective attention and reward (see Failing and Theeuwes 2018, for a review). An alternate explanation offered by additive factors logic (Sternberg 1969) is that the two expectations exert their influence at differing times in visual information processing. We find evidence that this indeed could be the case; whereas spatial certainty does influence priorities prior to target onset, the current data show that the influence of incentive value corresponds to counterfactual losses, which can only be known at the point of target and distractor onset. Critically, this account predicts that incentive values impact performance when there is a counterfactual loss, with negligible impact when the current outcome matches the best that could have happened.

Our observation that RTs are selectively slowed when participants must respond to a low value target location in the presence of a high value distractor accords with recent findings of selective slowing when high reward values are signalled at distractor locations relative to neutral target locations. For example, Watson et al (2020) showed that when participants are required to respond to a target in a visual search task that contains a single coloured distractor, participants are slower when that distractor signals high incentive value, relative to trials where a low incentive value is signalled instead. Moreover, Muller et al (2016) paired high or low incentive value cues with neutral colour cues in a dot probe task and again found selective slowing when the target appeared at a neutral location paired with a high value distractor, relative to trials where the low reward signal acted as a distractor instead. These findings have been interpreted as reflecting selective delayed disengagement of attentional mechanisms from stimuli that signal larger reward gains.

Our findings build on this work in the following ways; first, we provide new baseline conditions that can determine whether or not a high-value location was initially prioritised. We assume that this is relevant for attention disengagement accounts in the following way; if visual re-prioritisation is required to disengage from a high value location towards lower value locations, then prioritisation of the high value location must currently be higher relative to other locations. Therefore, there should be some behavioural benefit for prioritised locations relative to conditions where prioritisation between the locations is equal. Indeed, Watson et al (2020) showed that when a high incentive target is presented in a currently foveated location, RT performance is faster, relative to trials in which that foveated location signals low incentives. In the current work, we find that performance was comparable between high target value/low distractor value trials (hT/lD), and hT/hD trials (where we assume that prioritisation of either location should occur at chance). This suggests that incentive value does not always induce prioritisation of a high value location, therefore selective disengagement from a previously prioritised location may not always be sufficient to account for the influence of incentive value on performance.

One point of complement between the counterfactual and delayed disengagement accounts is that if there does exist a selective disengagement mechanism that is reflected only in changing disengagement durations (i.e. that is not the consequence of a previous prioritisation that benefitted performance), then perhaps the duration of disengagement can be modelled using such a counterfactual mechanism as we have investigated here. Importantly, in the current work we propose a quantitative computation that makes predictions regarding the extent to which selective slowing should be observed across multiple paradigms. However, this work is in its infancy and the extent to which the currently proposed counterfactual account is exclusive or complementary to other alternate accounts such as delayed disengagement needs to be determined in future studies. However, this is to our knowledge the first test showing that incentive value may act on a distinct stage of information processing to anticipatory expectations.

It remains to be determined to what extent previous observations of value driven visual prioritisation (e.g. Anderson, Laurent, and Yantis 2011; Itthipuripat et al. 2019; MacLean, Diaz, and Giesbrecht 2016) can also be thought of as reflecting a counterfactual loss signal. Indeed, a common feature of previous investigations is to present coloured cues that have been associated with incentive values in a new task under extinction, i.e. reward incentives are no longer available. The key finding is that participants show greater response slowing when the high value colour is presented as a distractor, relative to when the distractor is of low value or carries no value association. Given participants know they can no longer attain rewards in this stage, this observation has previously been interpreted as reflecting a competition between top-down settings and cue-reward associations. It is also possible that such competition reflects a counterfactual loss update. It may be beneficial to model what may have occurred given previous experiences with the incentive value cue (i.e. the reward learning phase), so that this cue can be prioritised should reward values become available again, or indeed to learn that this cue really no longer offers reward. Critically, a counterfactual account of incentive value cueing makes clear predictions about when in time such cues should influence visual information processing; specifically once the targets have onset and the current outcomes are known. This is in contrast to spatial certainty expectations, which are known to influence visual information processing prior to target onset (e.g. Zhigalov et al. 2019). We seek to follow up this clear and testable hypothesis in subsequent work.

It remains to be determined whether spatial certainty cues can always be best explained by anticipatory mechanisms, and incentive value cues counterfactual mechanisms, or whether task demands and cue formations can modify where and how each account best predicts performance. For example, in the current study (as well as many of the investigations into the influence of incentive value associations on visual prioritisation (Anderson, Laurent, and Yantis 2011; Garner, Bowman, and Raymond 2021; Itthipuripat et al. 2019; Kim and Anderson 2021; Le Pelley et al, 2015; Le Pelley et al, 2022; MacLean, Diaz, and Giesbrecht 2016; Stănişor et al. 2013; Stankevich and Geng 2014)), incentive value is not predictive of target location under the key testing conditions, and therefore offers no benefit for finding the target. It would be interesting to test within a single set of participants whether shifting between value cueing conditions that are predictive and non-predictive of upcoming target locations likewise shifts individuals between anticipatory and counterfactual styles of cue influence. Mapping anticipatory and counterfactual styles of responding to underlying neural signals will also be revealing. For example, recent work regarding the N2pc component during visual search shows that this component, which is tied to spatial shifts in visual processing, changes in latency with physical salience manipulations, and in amplitude with incentive value manipulations (Bachman et al. 2020). It may be that anticipatory and counterfactual styles of responding map to these dissociable influences on underlying neural activity. Future research should combine manipulations of physical salience, anticipatory predictions and counterfactual information to investigate this possibility.

It also remains to be determined to what extent the influence of incentive value cues remains independent to other types of spatial cue. For example, previous research does suggest an interactive relationship between exogenous spatial and incentive value cues under some conditions, for example when exogenous cues signal both a likely spatial location and a reward (Munneke, Hoppenbrouwers, and Theeuwes 2015). Although this suggests that value cues can tap into the action of exogenously driven visual prioritisations, other recent work suggests that responses to value cues may be distinct from exogenous mechanisms as their influence appears immune to the procedures that typically prevent distraction from physically salient stimuli (Pearson et al. 2020). Moreover, the current work focuses on the use of spatial and reward value information offered by separate cue signals. Previous work shows an interactive influence of dissociable information sources when they are both signalled by the very same cue; for example when a word cue signals both the upcoming spatial location of a target stimulus and the likely hand of response, yet an additive influence when separate cues are used (Mattler 2004). It remains to be determined whether combining spatial and incentive value information into a single cue would produce additive or interactive influences on responses, and whether such a combination would result in an anticipatory or counterfactual style of response.

Previous investigations into the neural representation of counterfactual outcomes have used overt choice paradigms (e.g. Boorman, Behrens, and Rushworth 2011; Fouragnan et al. 2019; Pischedda, Palminteri, and Coricelli 2020), where participants are instructed to select from between competing options. It is interesting that we observe evidence for a visual prioritisation signal that reflects counterfactual loss in the current paradigm, as participants have no choice over which reward outcome will be attained on any given trial. Specifically, they cannot choose where the target and distractor will appear. The current findings show that the brain still simulates counterfactual information even when this information cannot be used to inform future choices, which does accord with recent work showing that humans seek information that has no instrumental value (e.g. Brydevall et al. 2018; van Lieshout, de Lange, and Cools 2020). Here we show evidence that the visual system may model alternate outcomes even when those outcomes are non-instrumental, and that this information modifies visual priorities. Why might a system engage in such behaviour? We propose that counterfactual losses may act as a brake on the competition for visual representation; by detecting when evolving outcomes do not match what is desired, slowing behaviour may provide time for cues that predict desired outcomes to become more competitive for representation in awareness. Such a signal could act to put an agent back on track under circumstances where early deviations from the ideal outcome are tagged and addressed.

We found evidence for two subtypes of response to incentive values cues, however the interpretation of the two groups differs depending on the clustering algorithm used to detect them. Importantly, the same pattern of group differences were found regardless of the specific clustering algorithm used to organise the data into subtypes. Trackers used incentive value cues and spatial certainty cues and followers used only spatial certainty cues. This suggests that there is some weight to theoretical assertions that individuals will differ in how they leverage information to guide visual priorities (Tsotsos et al. 2021). However, what the current data show is that although some individuals may differ in which cues they use, it appears that how cues are encoded is consistent across individuals. This suggests that while individuals have some control over which sensory cues are used to guide their visual priorities, the nature of how they are encoded is set across individuals. Thus theories of motivated attention may need to account for how the individual’s motivational state may result in differing profiles of cue use, but not in how cues are used.

Interestingly, all individuals used spatial certainty cues suggesting that their use is compulsory. It remains to be determined whether this is because spatial cues are over-learned stimuli that invoke habitual responses (e.g. Lingawi, Dezfouli, and Balleine 2016; Dolan and Dayan 2013; Jiang and Sisk 2019), or if they reflect an automated hard-coded response to the spatial information inherent in the visual cue (Ristic and Kingstone 2012). Interestingly, although not tested formally the current data imply that participants who did not use incentive value cues were influenced to a greater extent by the central arrow cues that denoted spatial certainty. This could be because these participants were more susceptible to stimuli that invoke automated visual prioritisation, were unable to use both cues to modify priorities, or were unmotivated by the value cues which consequently left more leeway to be influenced by the spatial cue. We seek to address these possibilities in future studies.

As mentioned above, interpretation of the sources of subtypes changes depending on the clustering algorithm used to group the data. The more convincing clustering solution grouped participants as belonging to one homogeneous group, or as outliers that could not readily be assigned to any group. The statistical power required to perform a clustering analysis is largely dependent on the nature of clusters in the underlying data (Hair, Black, and Babin 2010). Given the novelty of the current research question, we opted to run a larger study (N = 149) to provide the best opportunity to identify qualitative subsets. However, it remains unknown whether the currently identified outliers genuinely reflect outliers or if they would attain membership to a new cluster given a sufficiently large number of data points. Moreover, although the statistically reliable behavioural differences between trackers and followers do validate the decision to accept a 2-cluster solution, it would be informative to further validate group membership on an external measure. For example, do followers suffer more cognitive failures in daily life than trackers? We seek to address these issues in future work.

## 6 Conclusions

There has been intense interest over the past decade in why incentive value cues may modify visual priorities, particularly when value cues are no more physically salient than other display items and such cues can be antithetical to the current task demands (Le Pelley et al, 2016; Chelazzi et al, L. Chelazzi et al. 2013; Raymond et al, 2009; Awh, Belopolsky, and Theeuwes 2012). Here we provide a clue for why incentive value cues act independently to other cues such as spatial cues which are assumed to be encoded into a priority map to guide visual priorities (Failing and Theeuwes 2018; Chelazzi et al, 2014; Awh, Belopolsky, and Theeuwes 2012). Specifically, we propose that incentive value cues are encoded into a representation of counterfactual loss that tracks how well current task outcomes map onto the best possible task outcomes given previous experiences with the same stimuli. Interestingly, although individuals differ in whether or not they engage with such cues, they are consistent in how they use them. Thus theoretical accounts of motivated attention may need to account for qualitatively different responses to reward associated cues. Our results imply that the role of incentive value in the visual system is to influence learning and adaptation; we propose that by slowing down performance in response to counterfactual losses the system gains time to boost the sensory representation of the best outcome given previous experience with elements of the visual scene.

## Data Accessibility Statement

The data is available at UQ eSpace (https://espace.library.uq.edu.au/view/UQ:d42fa1a). Custom code for the experimental task (https://github.com/kel-github/imaging-cert-reward-att-task-code) and analysis (https://github.com/kel-github/attention-clusters) are available on GitHub.

## Footnotes

https://doi.org/10.48610/d42fa1a.

https://github.com/kel-github/imaging-cert-reward-att-task-code and https://github.com/kel-github/attention-clusters.
